# DEC1 deficiency protects against bone loss induced by ovariectomy by inhibiting inflammation

**DOI:** 10.7555/JBR.38.20240069

**Published:** 2024-05-29

**Authors:** Lan Lin, Zhiyi Qiang, Kaiao Chen, Ying Huo, Wei Liu, Jian Yang

**Affiliations:** Department of Pharmacology, Nanjing Medical University, Nanjing, Jiangsu 211166, China

**Keywords:** DEC1, postmenopausal osteoporosis, estrogen, osteoblasts, osteoclasts

## Abstract

Studies have shown that differentiated embryo-chondrocyte expressed gene 1 (DEC1) promotes osteoblast osteogenesis. To investigate the role of DEC1 in postmenopausal osteoporosis, we used the two genotypes of mice ( *Dec1* ^+/+^ and *Dec1* ^−/−^) to establish an ovariectomy model and found that the bone loss was significantly lower in *Dec1* ^−/−^ ovariectomy mice than in *Dec1* ^+/+^ ovariectomy mice. The expression levels of RUNX2 and OSX were significantly increased in *Dec1* ^−/−^ ovariectomy mice, compared with *Dec1* ^+/+^ ovariectomy mice; however, the expression levels of NFATc1, c-Fos, CTSK, and RANKL/OPG ratio were significantly decreased in *Dec1* ^−/−^ ovariectomy mice, compared with those in *Dec1* ^+/+^ ovariectomy mice. Likewise, DEC1 deficiency also suppressed the expression levels of IL-6 and IL-1β. Further results showed that the mRNA expression levels of *Runx2*, *Osx*, and *Alp* were significantly increased in bone marrow mesenchymal stem cells of *Dec1* ^−/−^ ovariectomy mice, compared with those of *Dec1* ^+/+^ ovariectomy mice. Moreover, the mRNA levels of *Il1b*, *Il6*, *Tnfa*, and *Ifng* were significantly increased in bone marrow-derived macrophages (BMMs) of *Dec1* ^+/+^ovariectomy mice, compared with those of *Dec1* ^+/+^ sham mice, but not in *Dec1* ^−/−^ ovariectomy BMMs, when compared with those in *Dec1* ^−/−^ sham BMMs. Additionally, the expression levels of p-IκBα and p-P65 were significantly increased in *Dec1* ^+/+^ ovariectomy BMMs, compared with those in *Dec1* ^+/+^ sham BMMs, but did not increase in *Dec1* ^−/−^ ovariectomy BMMs, compared with those in *Dec1* ^−/−^ sham BMMs. Taken together, DEC1 deficiency inhibited the NF-κB pathway induced by ovariectomy, thereby decreasing cytokines and subsequently inhibiting the decrease of osteogenesis and the increase of osteoclastogenesis caused by ovariectomy. The findings may provide a novel understanding of postmenopausal osteoporosis development, and offer potential avenues for the disease intervention.

## Introduction

Postmenopausal osteoporosis (PMOP) is a bone metabolic disease that results in reduced bone mass and the destruction of bone tissue microstructure ^[ 1] ^. It primarily affects middle-aged and elderly women, representing the most prevalent form of osteoporosis in this demographic. Statistically, approximately 50% of women sustain at least one fracture after menopause ^[ 2] ^. Because of the aging global population, there has been a significant rise in the number of patients suffering from postmenopausal osteoporosis, resulting in a substantial demand for medical resources. PMOP is caused by the decline of ovarian function after menopause and the sharp drop in estrogen levels in the body, leading to an imbalance in bone remodeling. Bone remodeling is a strictly regulated process that involves bone resorption mediated by osteoclasts and bone formation mediated by osteoblasts, with a dynamic balance between bone resorption and bone formation ^[ 3– 4] ^. Consequently, it is of paramount importance to elucidate the precise mechanisms underlying bone remodeling after PMOP. The bilateral ovariectomy (OVX) mouse model, a classical model for simulating postmenopausal osteoporosis, is extensively used to analyze the mechanism of estrogen-deficient osteoporotic diseases ^[ 5] ^. First, OVX was found to affect the stabilization of β-catenin through the down-regulation of the classical Wnt/β-catenin pathway, which in turn downregulated the expression of both runt-related transcription factor 2 (RUNX2) and osterix (OSX) that were involved in osteoblast differentiation, maturation, and activity ^[ 6– 7] ^. Likewise, OVX also activated the NF-κB pathway in osteocytes and induced osteocyte apoptosis ^[ 8] ^. Second, OVX enhanced osteoclast differentiation and accelerated bone resorption in mice ^[ 9– 10] ^. The increased activity of osteoclasts induced by OVX occurred through the receptor activator of nuclear factor-kappaB (NF-κB) ligand (RANKL)-mediated NF-κB and nuclear factor of activated T-cells 1 (NFATc1) pathways ^[ 11] ^. The RANKL was reported to bind to the receptor activator of NF-κB (RANK) and then activate the key osteoclast regulators, such as NFATc1, c-Fos, and CTSK, to promote the osteoclast differentiation ^[ 12– 14] ^. Third, the differentiated osteoblasts also produced both positive and negative regulators of osteoclastogenesis, including RANKL and its natural decoy receptor osteoprotegerin (OPG) ^[ 15] ^. Estrogen deficiency upregulated the expression of RANKL but downregulated the expression of OPG, resulting in an increase in the RANKL/OPG ratio ^[ 16] ^. Fourth, the withdrawal of estrogen was associated with a spontaneous increase in pro-inflammatory cytokines, such as TNF-α, IL-1β, and IFN-γ, that stimulated bone resorption ^[ 17] ^. Overall, OVX hinders osteogenesis and facilitates the development of osteoclastogenesis, leading to bone loss.

Differentiated embryo-chondrocyte expressed gene 1 (DEC1) is a structurally unique basic helix-loop-helix protein that is a major regulator of a variety of physiological and pathological processes, including circadian rhythms, hypoxia, cellular proliferation, apoptosis, immunity, and inflammation ^[ 18– 22] ^. Initially identified in human chondrocytes, DEC1 is closely linked to bone growth and remodeling ^[ 23] ^. We previously found that DEC1 enhanced osteoblast osteogenesis and that icariin, which upregulated DEC1 expression in SaoS2 cells, might protect against glucocorticoid-induced osteoporosis ^[ 24] ^. These studies imply that DEC1 is a crucial factor in the formation of osteoblasts. Furthermore, our previous study also found a decrease in DEC1 in the bone tissue of OVX mice ^[ 25] ^. However, the role of DEC1 in PMOP remains to be determined.

In the current study, we used two genotypes ( *Dec1* ^+/+^ and *Dec1* ^−/−^) of mice to establish an OVX model and determine the effects and mechanisms of DEC1 in PMOP.

## Materials and methods

### Animals

The heterozygous *Dec1* ( *Dec1* ^+/−^) C57BL/6 mice were purchased from the RIKEN BioResource Research Center in Japan and bred at the Animal Core Facility of Nanjing Medical University. *Dec1* heterozygous ( *Dec1* ^+/−^) C57BL/6 mice were mated to obtain offspring, from which *Dec1* gene knockout ( *Dec1* ^−/−^) mice and wild-type ( *Dec1* ^+/+^) mice were selected from the littermates. Double checks (after birth and before the experiment) were applied to ensure the correct mouse genotype. The mouse genotype was confirmed as shown in ***Supplementary Fig. 1A*** (available online). Mice were housed in a specific pathogen-free barrier environment with good ventilation, a 12 h∶12 h light-dark cycle, temperature maintained at 22–26 ℃, and humidity kept constant at 50% to 60%, with free access to water and food. Efforts were made to minimize animal suffering and to reduce the number of animals used for the experiments. All the animal experiments were strictly in compliance with the experimental animal guidelines of the Laboratory Animal Research Institute and were approved by the Animal Ethical and Welfare Committee of Nanjing Medical University (IACUC-2203015).

### Establishment of ovariectomy mouse models

OVX mouse models were established according to a previously reported study with minor modifications ^[ 26] ^. Both 20 *Dec1* ^+/+^ mice and 20 *Dec1* ^−/−^ mice, aged 6–8 weeks, were randomly divided into the sham and OVX groups, with 10 mice in each group. After intraperitoneal injection of 4% chloral hydrate (0.01 mL/10 g) for anesthesia, the mice in the OVX group were ovariectomized bilaterally, and the corresponding control mice were sham-operated (sham).

### BMSCs and BMMs were isolated and cultured from the OVX mice

One month after the operation, the mice of *Dec1* ^+/+^ sham, *Dec1* ^+/+^ OVX, *Dec1* ^−/−^ sham, and *Dec1* ^−/−^ OVX were euthanized, and the tibiae and femurs were aseptically dissected to collect bone marrow cells. The adherent cells were cultured until reaching a confluence of 80%. BMSCs from passages five to nine were harvested for subsequent experiments. BMMs from the mice were cultured in alpha-MEM medium (Cat. #12571071, Gibco, Grand Island, NY, USA) supplemented with 5 ng/mL macrophage colony-stimulating factor (M-CSF; Cat. #315-02, PeproTech, Rocky Hill, NJ, USA), 1% penicillin/streptomycin, and 10% fetal bovine serum (FBS; Cat. #086-150, Wisent, Saint-Jean-Baptiste, QC, Canada). Both BMSCs and BMMs from the two groups of mice ( *Dec1* ^+/+^ sham and *Dec1* ^+/+^ OVX) or four groups of mice ( *Dec1* ^+/+^ sham, *Dec1* ^+/+^ OVX, *Dec1* ^−/−^ sham, and *Dec1* ^−/−^ OVX) were used in subsequent experiments.

### Micro-CT analysis

The femurs of mice were fixed overnight in 4% paraformaldehyde and subjected to the micro-CT analysis using a SkyScan scanner (SkyScan1172, Bruker, Kontich, Belgium) with a scanning precision of 18 μm. The obtained scan data were reconstructed in three dimensions using the CTvox software. Bone trabecular and cortical bone parameters, including bone mineral density (BMD, mg/cc), bone volume fraction (BV/TV), trabecular number (Tb.N, per mm), trabecular thickness (Tb.Th, μm), trabecular separation (Tb.Sp, mm), total area (Tt.Ar, mm ^2^), cortical bone area (Ct.Ar, mm ^2^), and cortical bone thickness (Ct.Th, mm), were analyzed.

### Morphometric analysis of bone tissues

The femurs of five-month-old mice were fixed in 4% paraformaldehyde for 24 h, followed by decalcification in 10% EDTA for two weeks. Subsequently, the bones were dehydrated in ethanol and xylene, embedded in paraffin, and sectioned into 5-μm thick slices using a paraffin microtome (RM2245, Leica, Wetzlar, Germany). The bone sections were stained with hematoxylin and eosin (HE) staining (Cat. #D006-1-4, Nanjing Jiancheng Bioengineering Institute, Nanjing, Jiangsu, China).

### Immunohistochemistry (IHC) staining

Sections were dewaxed in xylene and then rehydrated through an ethanol concentration gradient. Tissue samples were then deactivated with 3% H _2_O _2_. Antigen retrieval was performed using trypsin (Cat. #T8150, Solarbio, Beijing, China), followed by blocking the sections with 5% goat serum (Cat. #AR0009, BOSTER, Pleasanton, CA, USA) for one hour. Subsequently, sections were incubated overnight at 4 ℃ with specific antibodies against β-catenin (1∶500, Cat. #PK02151, Abmart, Shanghai, China), RUNX2 (1∶200, Cat. #sc-390351, Santa Cruz Biotechnology, Dallas, TX, USA), OSX (1∶500, Cat. #ab209484, Abcam, Cambridge, UK), OPG (1∶500, Cat. #DF6824, Affinity, Cincinnati, OH, USA), NFATc1 (1∶200, Cat. #sc-7294, Santa Cruz Biotechnology), c-Fos (1∶500, Cat. #66590-1-Ig, Proteintech, Rosemont, IL, USA), CTSK (1∶200, Cat. #sc-48353, Santa Cruz Biotechnology), and RANKL (1∶500, Cat. #bs-20647R, Bioss Antibodies, Woburn, MA, USA). Then, the sections were incubated with HRP-conjugated secondary antibodies at room temperature for one hour, followed by counterstaining with hematoxylin and sealing with neutral resin. Immunoreactivity was detected using an optical microscope (BX53, Olympus, Tokyo, Japan).

### Western blotting analysis

For the extraction of protein from bone tissues, the femur was frozen in liquid nitrogen for 15 to 30 min, and then crushed and homogenized in RIPA lysis buffer (Cat. #C1053, APPLYGEN, Beijing, China). The homogenate was then centrifuged at 12000 *g* and 4 ℃ for 15 min. The supernatant was collected, and protein concentrations were determined with the BCA protein assay (Pierce Chemical, Dallas, TX, USA) based on albumin standard. The proteins were subjected to SDS-PAGE, and then transferred onto a PVDF membrane. The membrane was blocked with 5% BSA and incubated overnight at 4 ℃ with specific primary antibodies as follows: β-catenin (1∶1000, Cat. #PK02151, Abmart), RUNX2 (1∶500, Cat. #sc-390351, Santa Cruz Biotechnology), OSX (1∶1000, Cat. #ab209484, Abcam), OPG (1∶1000, Cat. #DF6824, Affinity Biosciences), NFATc1 (1∶500, Cat. #sc-7294, Santa Cruz Biotechnology), c-Fos (1∶1000, Cat. #66590-1-Ig, Proteintech), CTSK (1∶500, Cat. #sc-48353, Santa Cruz Biotechnology), RANKL (1∶1000, Cat. #bs-20647R, Bioss Antibodies), DEC1 (1∶500, Cat. #sc-101023, Santa Cruz Biotechnology), Phospho-NF-κB p65 (Ser536) (1∶1000, Cat. #3033, Cell Signaling Technology), NF-κB p65 (1∶1000, Cat. #8242, Cell Signaling Technology), Phospho-IκBα (Ser32) (1∶1000, Cat. #2859, Cell Signaling Technology), IκBα (1∶1000, Cat. #9242, Cell Signaling Technology), matrix metallopeptidase 9 (MMP9; 1∶1000, Cat. #10375-2-AP, Proteintech), and β-actin (1∶1000, Cat. #AP0060, Bioworld Technology, Nanjing, China). After washing with TBST, the PVDF membrane was incubated with an HRP-conjugated secondary antibody at room temperature for one hour. Following the manufacturer's instructions, protein bands were detected using the ECL Western blotting detection system (Cat. #E423-01/02, Vazyme, Nanjing, China). Chemiluminescent signals were captured using image analysis software (ImageJ, 1.54i, NIH). β-Actin was used as an internal control.

### Enzyme-linked immunosorbent assay (ELISA)

Mouse serum was collected from extracted eyeball blood. Blood samples were allowed to clot at room temperature for 30 min and then stored at 4 ℃ for an additional 30 min. The samples were centrifuged at 3000 *g* and 4 ℃ for 10 min to obtain the serum. The concentrations of estradiol (Cat. #AF2566-A, AiFang Biological, Changsha, China), CTX-1 (Cat. #AF2808-A, AiFang Biological), IL-1β (Cat. #AF2040-A, AiFang Biological), IL-6 (Cat. #AF2163-A, AiFang Biological), and TNF-α (Cat. #KE10002, Proteintech) in the serum were determined by using an ELISA kit.

### RNA isolation and real-time reverse transcription-PCR (RT-qPCR)

Total RNA was isolated from cultured cells using TRIzol reagent (Cat. #15596018CN, Thermo Fisher Scientific, Waltham, MA, USA), followed by cDNA synthesis using a reverse transcription kit (Cat. #R222-01, Vazyme). RT-qPCR was performed using the FastStar Universal SYBR Green Master (Cat. #Q311-02/03, Vazyme) and the 7300 real-time PCR system (Applied Biosystems, Foster City, CA, USA). β-Actin was used as an internal control for cDNA. Primer sequences are listed in ***Table 1***.

**1:** Primers used in real-time reverse transcription-PCR

Genes	Forward (5′–3′)	Reverse (5′–3′)
*Runx2*	AGTAGCCAGGTTCAACGATCTGA	GACTGTTATGGTCAAGGTGAAACTCTT
*Osx*	TTCTGTCCCCTGCTCCTTCTAG	CGTCAACGACGTTATGCTCTTC
*Alp*	AACACCAATGTAGCCAAG	TCGGGCAGCGGTTACTGT
*Ocn*	CCAAGCAGGAGGGCAATA	AGGGCAGCACAGGTCCTAA
*Rankl*	AGCGCAGATGGATCCTAACA	CCAGAGTCGAGTCCTGCAAAT
*Opg*	GTGGAATAGATGTCACCCTGTGT	TTTGGTCCCAGGCAAACTGT
*Nfatc1*	GACCCGGAGTTCGACTTCG	TGACACTAGGGGACACATAACTG
*c-Fos*	CGGGTTTCAACGCCGACTA	TTGGCACTAGAGACGGACAGA
*Ctsk*	GAAGAAGACTCACCAGAAGCAG	TCCAGGTTATGGGCAGAGATT
*Trap*	CACTCCCACCCTGAGATTTGT	CATCGTCTGCACGGTTCTG
*Il1b*	CAACCAACAAGTGATATTCTCCATG	GATCCACACTCTCCAGCTGCA
*Il6*	GAGGATACCACTCCCAACAGACC	AAGTGCATCATCGTTGTTCATACA
*Tnfa*	CATCTTCTCAAAATTCGAGTGACAA	TGGGAGTAGACAAGGTACAACCC
*Ifng*	ATGAACGCTACACACTGCATC	CCATCCTTTTGCCAGTTCCTC
*Actb*	GGCTGTATTCCCCTCCATCG	CCAGTTGGTAACAATGCCATGT

### Statistical analysis

All the data were expressed as mean ± standard deviation. Statistical analysis for multiple groups was performed using GraphPad Prism (GraphPad Prism, version 9, San Diego, CA, USA). Student's *t*-test or two-way ANOVA followed by Tukey's post hoc tests were performed to compare the differences between two groups or among more than two groups. Statistical significance was considered at a *P*-value of less than 0.05 ( *P* < 0.05).

## Results

### DEC1 deficiency primarily reduced trabecular bone loss caused by OVX in mice

*Dec1* knockout C57BL/6 mice ( *Dec1* ^−/−^) and littermate wild-type mice ( *Dec1* ^+/+^) were used to establish the OVX mice whose pathological features were similar to PMOP ^[ 5] ^. Estradiol is one of the most common and crucial estrogens. As shown in ***Supplementary Fig. 1***, levels of the serum estradiol were significantly reduced in both *Dec1* ^+/+^ and *Dec1* ^−/−^ mice three months post bilateral OVX operation, compared with those in the corresponding sham mice ( ***Supplementary Fig. 1B***). The weight was significantly increased in both *Dec1* ^+/+^ and *Dec1* ^−/−^ OVX mice, compared with that in the corresponding sham mice ( ***Supplementary Fig. 1C***). The size of the uterus in *Dec1* ^+/+^ OVX mice, rather than in *Dec1* ^−/−^ OVX mice, significantly decreased compared with that in corresponding sham mice ( ***Supplementary Fig. 2A*** [available online]). The reason for no significant change in the uterus between the OVX and sham of *Dec1* ^−/−^ mice might be that the ERα expression levels of the uterus were increased in *Dec1* ^−/−^ OVX mice than in *Dec1* ^+/+^ OVX mice ( ***Supplementary Fig. 2B***– *2D*). However, the expression levels of serum type I collagen C-terminal telopeptide (CTX-1), a byproduct of bone remodeling ^[ 27] ^, were significantly increased in *Dec1* ^+/+^ OVX mice than in *Dec1* ^+/+^ sham mice, whereas the serum CTX-1 levels were not significantly different between *Dec1* ^−/−^ OVX mice and *Dec1* ^−/−^ sham mice ( ***Supplementary Fig. 1D***). The results suggested that the OVX model was established in both *Dec1* ^+/+^ and *Dec1* ^−/−^ mice, and that bone turnover was increased in *Dec1* ^+/+^ OVX mice but not in *Dec1* ^−/−^ OVX mice.

**Figure 1:**
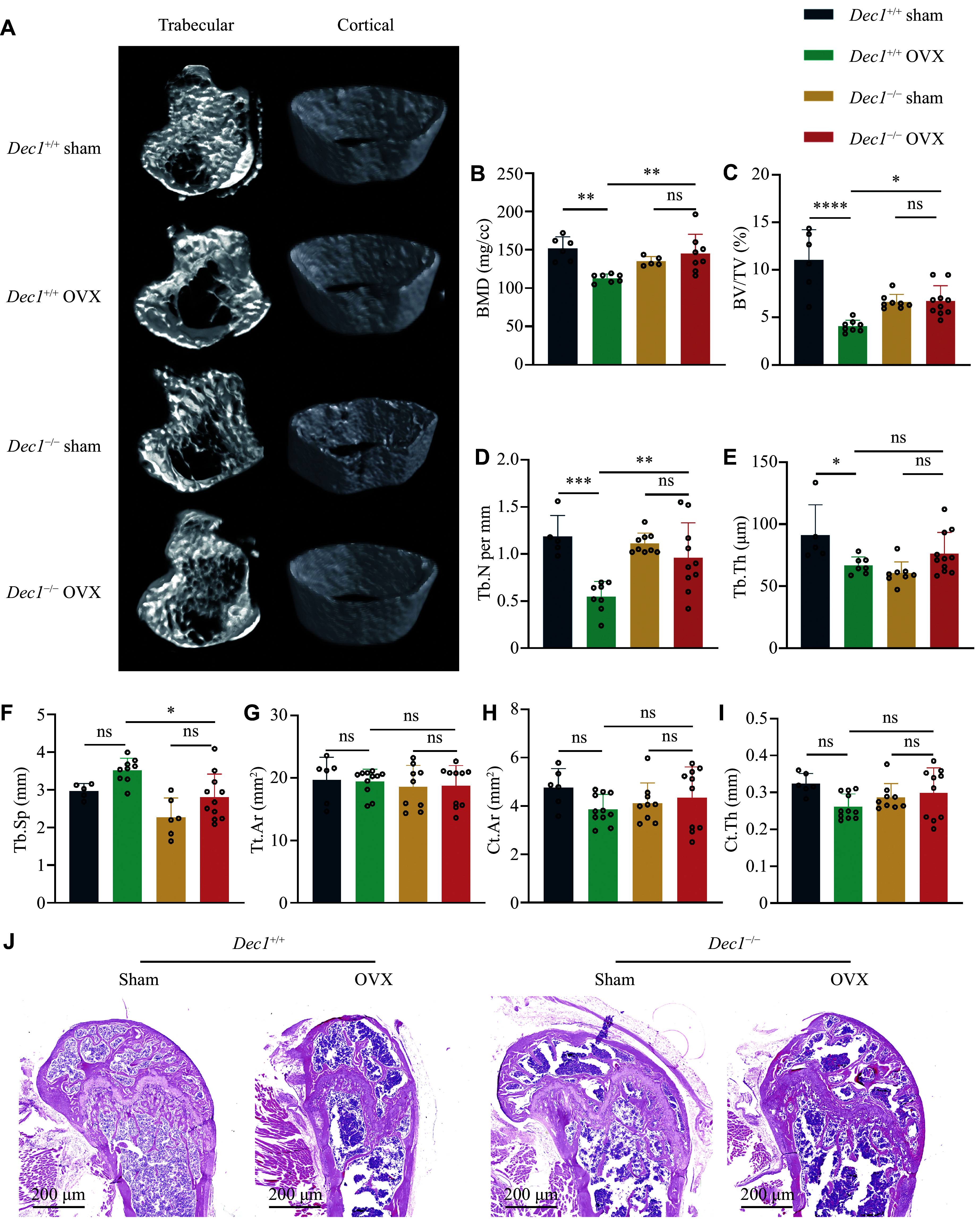
DEC1 deficiency primarily reduced trabecular bone loss caused by OVX in mice. A: Representative images of three-dimensional reconstruction of mouse femoral trabecular and cortical bone. B: The mean bone mineral density (BMD) value within each group ( *n* = 6–8 in each group). C–I: Micro-CT analysis of bone volume/trabecular bone volume ratio (BV/TV) (C), trabecular bone number (Tb.N) (D), trabecular bone thickness (Tb.Th) (E), trabecular spacing (Tb.Sp) (F), total area (Tt.Ar) (G), cortical area (Ct.Ar) (H), and cortical thickness (Ct.Th) (I) of the distal femur metaphysis ( *n* = 5–12 in each group). J: Representative images of H&E staining of the femur in each group of mice ( *n* = 5–12 in each group). Data are presented as mean ± standard deviation and analyzed using two-way ANOVA followed by Tukey's honestly significant difference tests. ^*^ *P* < 0.05, ^**^ *P* < 0.01, ^***^ *P* < 0.001, and ^ns^ *P* > 0.05. Abbreviations: DEC1, differentiated embryo-chondrocyte expressed gene 1; OVX, ovariectomy.

Next, we performed micro-CT scans on the femurs of the mice and conducted quantitative analyses of bone parameters to reveal differences in the microarchitecture of the bones among the four groups of mice ( *Dec1* ^+/+^ sham, *Dec1* ^+/+^ OVX, *Dec1* ^−/−^ sham, and *Dec1* ^−/−^ OVX). As shown in ***Fig. 1***, the bone mineral density of the trabecular bone of the femur was significantly lower in *Dec1* ^+/+^ OVX mice than in *Dec1* ^+/+^ sham mice, as indicated by the representative micro-CT reconstruction; however, this difference was not found between *Dec1* ^−/−^ OVX mice and *Dec1* ^−/−^ sham mice ( ***Fig. 1A***, left lane). Furthermore, there was no significant difference in cortical bone among the four groups ( ***Fig. 1A***, right lane). The results of the micro-CT quantitative analysis showed that in *Dec1* ^+/+^ OVX mice, there was a significant decrease in BMD, BV/TV, Tb.N, and Tb.Th, but a slight increase in Tb.Sp, compared with that in *Dec1* ^+/+^ sham mice. However, there were no significant differences in these bone mass parameters between *Dec1* ^−/−^ OVX and *Dec1* ^−/−^ sham mice ( ***Fig. 1B***– ***1F***). Moreover, the bone mass parameters, including BMD, BV/TV, Tb.N, and Tb.Sp, in *Dec1* ^−/−^ OVX mice were significantly increased, compared with those in the *Dec1* ^+/+^ OVX group ( ***Fig. 1B***– ***1D*** and ***Fig. 1F***). Notably, there was no significant difference in the cortical bone of the femur, including Tt.Ar, Ct.Ar, and Ct.Th, among the four groups ( ***Fig. 1G***– ***1I***). These changes were further demonstrated by H&E staining ( ***Fig. 1J***). These results implied that DEC1 deficiency primarily reduced trabecular bone loss caused by OVX in mice.

### DEC1 deficiency mitigated the diminished osteogenic activity induced by OVX in mice

**Figure 2:**
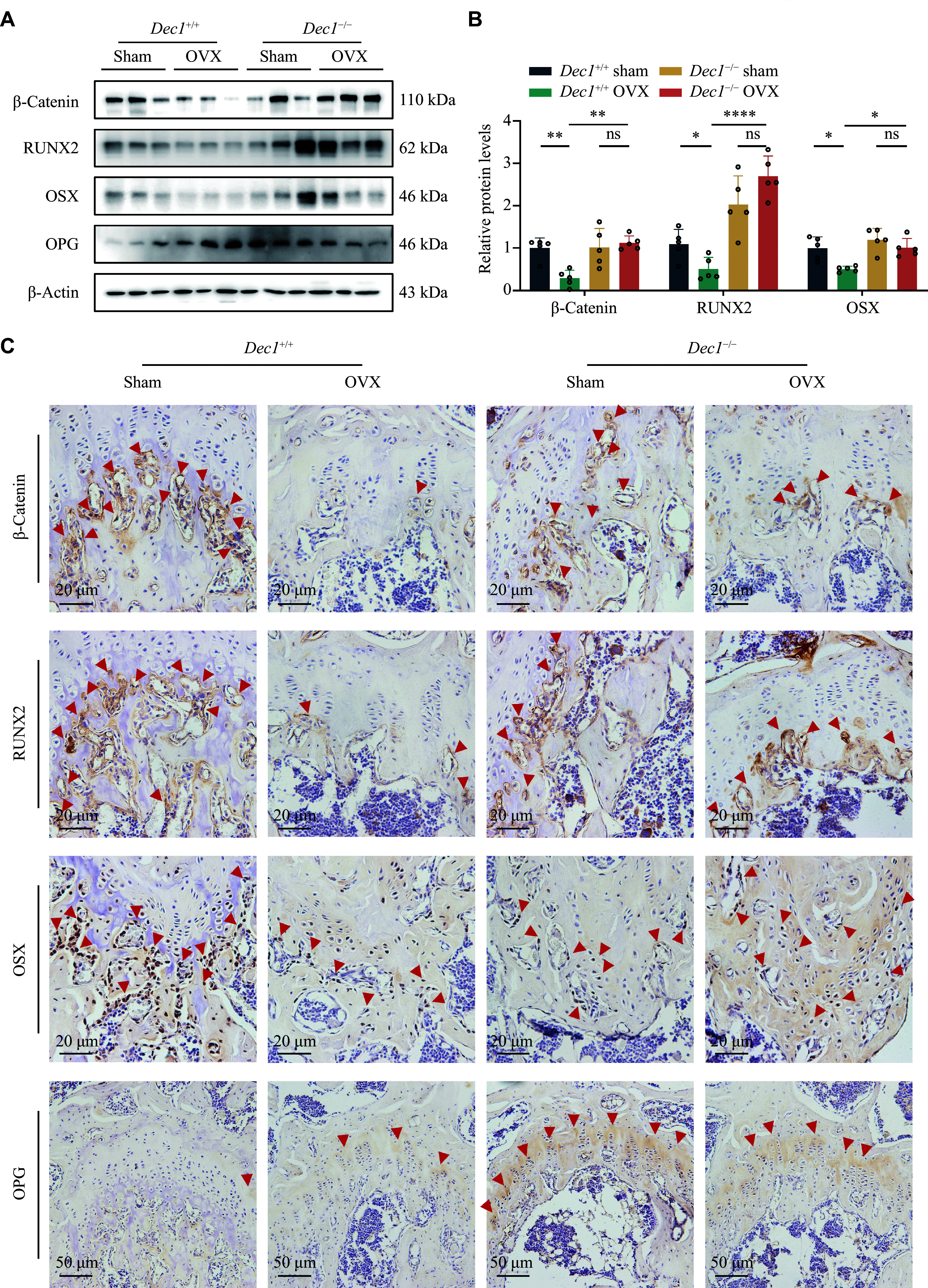
DEC1 deficiency mitigated the diminished osteogenic activity induced by OVX in mice. A and B: The protein levels of β-catenin, RUNX2, OSX, and OPG in the femur in the two genotypes of OVX mice by Western blotting analysis ( *n* = 5 in each group). C: The protein levels of β-catenin, RUNX2, OSX, and OPG in the femur of the four groups of mice by immunostaining analysis. Scale bar, 20 μm or 50 μm ( *n* = 5 in each group). Red arrowheads indicate the positive stainings in immunohistochemistry. Data are presented as mean ± standard deviation and analyzed using two-way ANOVA followed by Tukey's honestly significant difference tests. ^*^ *P* < 0.05, ^**^ *P* < 0.01, ^****^ *P* < 0.0001, and ^ns^ *P* > 0.05. Abbreviations: DEC1, differentiated embryo-chondrocyte expressed gene 1; OVX, ovariectomy; RUNX2, runt-related transcription factor 2; OSX, osterix; OPG, osteoprotegerin.

To determine the effect of *Dec1* knockout on osteogenesis in mice, we examined the key proteins related to the differentiation and activity of osteoblasts, including β-catenin, RUNX2, OSX, and OPG, in the femur of the two genotypes of OVX mice by Western blotting. As shown in ***Fig. 2A*** and ***2B***, the expression levels of β-catenin, RUNX2, and OSX were significantly lower in *Dec1* ^+/+^ OVX mice than in *Dec1* ^+/+^ sham mice, while these proteins showed no significant difference between *Dec1* ^−/−^ OVX and *Dec1* ^−/−^ sham mice. Consistently, the IHC staining results showed that β-catenin, RUNX2, and OSX exhibited similar alterations in osteoblasts at the edge of trabeculae ( ***Fig. 2C***). Meanwhile, as a secretory protein, OPG was uniformly distributed in the growth plate. The OPG expression levels in the femoral growth plate were increased in *Dec1* ^−/−^ OVX mice, compared with those in *Dec1* ^+/+^ OVX mice ( ***Fig. 2C***). These results indicated that *Dec1* knockout alleviated the diminished osteogenic activity induced by OVX in mice.

### DEC1 deficiency alleviated the enhanced bone resorption induced by OVX in mice

**Figure 3:**
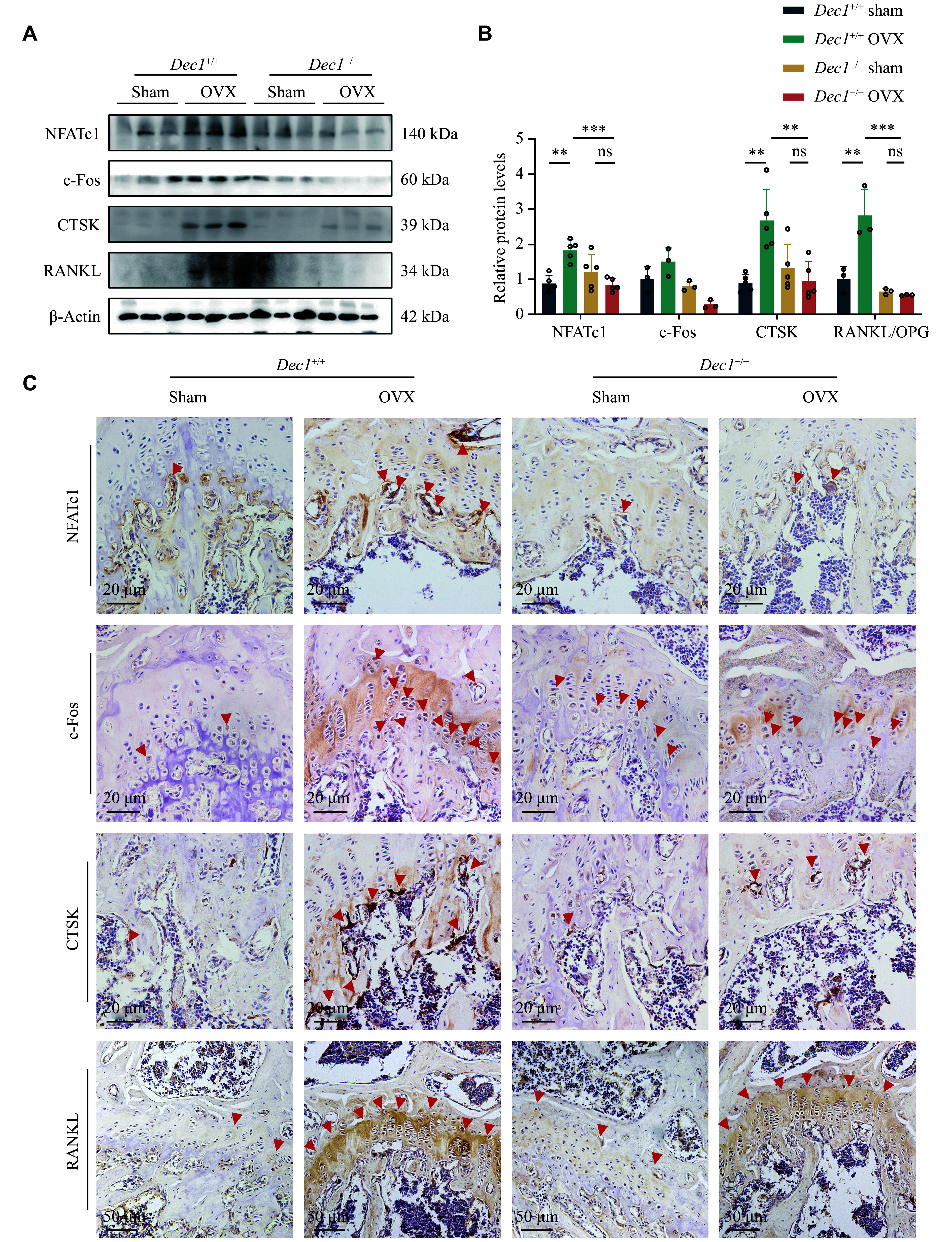
A and B: The protein levels of NFATc1, c-Fos, CTSK, and RANKL in the femur in the two types of OVX mice by Western blotting analysis ( *n* = 5 in each group). C: The protein levels of NFATc1, c-Fos, CTSK, and RANKL in the femur in the two types of OVX mice by immunostaining analysis. Scale bar, 20 μm or 50 μm. Red arrowheads indicate the positive stainings in immunohistochemistry. Data are presented as mean ± standard deviation and analyzed using two-way ANOVA followed by Tukey's honestly significant difference tests. ^**^ *P* < 0.01, ^***^ *P* < 0.001, and ^ns^ *P* > 0.05. Abbreviations: DEC1, differentiated embryo-chondrocyte expressed gene 1; OVX, ovariectomy; NFATc1, nuclear factor of activated T cells 1; CTSK, cathepsin K; RANKL, receptor activator of nuclear factor kappa-B ligand.

We analyzed the expression of osteoclast-specific genes to elucidate the effect of DEC1 on the formation and function of osteoclasts induced by OVX. The Western blotting results showed that the expression levels of RANKL were significantly increased in *Dec1* ^+/+^ OVX mice, compared with those in *Dec1* ^+/+^ sham mice, whereas it did not significantly increase in *Dec1* ^−/−^ OVX mice, compared with those in *Dec1* ^−/−^ sham mice ( ***Fig. 3A***). Furthermore, the results of IHC staining showed that RANKL was secreted into the growth plate ( ***Fig. 3C***). Combined with the fourth line of ***Fig. 2A*** and the fourth line of ***Fig. 3A***, we observed that the RANKL/OPG ratio was significantly increased in *Dec1* ^+/+^ OVX mice, compared with that in *Dec1* ^+/+^ sham mice, but not in *Dec1* ^−/−^ OVX mice, compared with that in *Dec1* ^−/−^ sham mice ( ***Fig. 3B***). In addition, the protein levels of NFATc1, c-Fos, and CTSK were significantly increased in *Dec1* ^+/+^ OVX mice, compared with those in *Dec1* ^+/+^ sham mice, while these indicators did not increase in *Dec1* ^−/−^ OVX mice, compared with those in *Dec1* ^−/−^ sham mice ( ***Fig. 3A*** and ***3B***). IHC results revealed consistent protein levels of NFATc1, c-Fos, and CTSK in osteoclasts at the edge of trabeculae ( ***Fig. 3C***). These results demonstrated that *Dec1* knockout alleviated the enhanced bone resorption induced by OVX in mice.

### DEC1 deficiency mitigated the increased inflammation induced by OVX in mice

**Figure 4:**
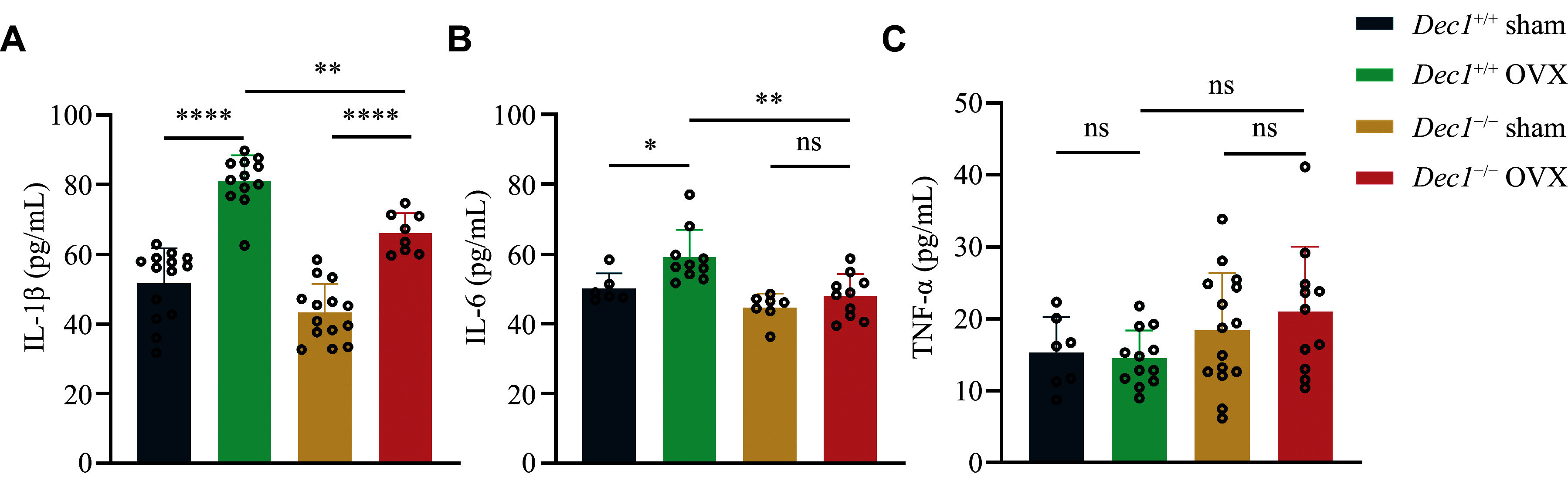
DEC1 deficiency mitigated the increased inflammation induced by OVX in mice. A–C: The serum amounts of IL-1β, IL-6, and TNF-α by enzyme-linked immunosorbent assay in the two genotypes of OVX mice ( *n* = 6–14 in each group). Data are presented as mean ± standard deviation and analyzed using two-way ANOVA followed by Tukey's honestly significant difference tests. ^*^ *P* < 0.05, ^**^ *P* < 0.01, ^****^ *P* < 0.0001, and ^ns^ *P* > 0.05. Abbreviations: DEC1, differentiated embryo-chondrocyte expressed gene 1; OVX, ovariectomy; ILs, interleukins; TNF-α, tumor necrosis factor-α.

OVX in mice leads to decreased estrogen levels, resulting in an increase in systemic inflammatory cytokines ^[ 28] ^. Inflammatory cytokines, such as IL-1β and TNF-α, have long been implicated in osteoblastic bone loss. These cytokines promote the production of RANKL by both osteoblast precursor cells and mature osteoblasts, and also reduce OPG production ^[ 29] ^. To investigate the role of DEC1 in the inflammatory process induced by OVX, we collected the serum from the orbital blood of model mice for ELISA detection. As shown in ***Fig. 4***, the serum levels of IL-1β and IL-6, but not TNF-α, were significantly increased in *Dec1* ^+/+^ OVX mice, compared with those in *Dec1* ^+/+^ sham mice. Although IL-1β levels were increased in both *Dec1* ^+/+^ and *Dec1* ^−/−^ OVX mice, compared with those in corresponding sham mice, the serum levels of IL-1β and IL-6, but not TNF-α, significantly decreased in *Dec1* ^−/−^ OVX mice, compared with those in *Dec1* ^+/+^ OVX mice. These data indicated that *Dec1* deletion inhibited the increased inflammation induced by OVX.

### Decreased DEC1 in BMSCs and increased DEC1 in BMMs from *Dec1* ^+/+^OVX mice

BMSCs are pluripotent stem cells with self-renewal and multidirectional differentiation capabilities. As a vital source of osteoprogenitor cells, BMSCs are crucial for maintaining normal physiological functions of bone tissues, and promoting bone repair and regeneration ^[ 30] ^. BMMs are a type of mononuclear cells with the potential to differentiate into various cell types ^[ 31] ^. When stimulated by factors such as RANKL and M-CSF, BMMs may differentiate into osteoclasts ^[ 32] ^.

**Figure 5:**
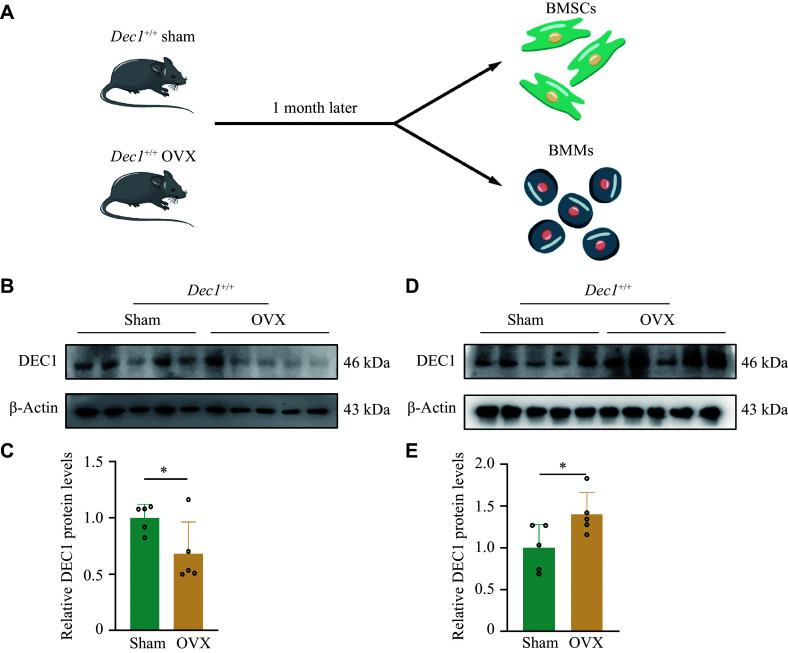
The DEC1 expression was decreased in BMSCs but increased in BMMs from the wild-genotype OVX mice compared with that of the sham mice. A: The flow chart of isolating BMSCs and BMMs from the wild-type OVX mice ( *n* = 5 in each group). B and C: DEC1 expression in BMSCs in *Dec1* ^+/+^ mice by Western blotting analysis ( *n* = 5 in each group). D and E: DEC1 expression in BMMs in *Dec1* ^+/+^ mice by Western blotting analysis ( *n* = 5 in each group). Data are presented as mean ± standard deviation and analyzed using Student's *t*-test. ^*^ *P* < 0.05. Abbreviations: DEC1, differentiated embryo-chondrocyte expressed gene 1; BMSCs, bone marrow mesenchymal stem cells; BMMs, bone marrow-derived macrophages; OVX, ovariectomy.

The heterogeneity of DEC1 expression in cells has significant implications for various biological processes. To investigate the DEC1 differential expression in BMSCs and BMMs, we isolated and cultured the two types of cells from *Dec1* ^+/+^ OVX or sham mice, respectively ( ***Fig. 5A***). We found that the protein levels of DEC1 were significantly decreased in BMSCs but increased in BMMs from *Dec1* ^+/+^ OVX mice, compared with those from *Dec1* ^+/+^ sham mice ( ***Fig. 5B***– ***5E***). These results indicated that the effects of OVX on DEC1 expression in BMSCs and BMMs of *Dec1* ^+/+^ mice were opposite.

### DEC1 deficiency enhanced osteoblast activity along with attenuating the NF-κB pathway and increasing the RANKL/OPG ratio in BMSCs from the OVX mice

**Figure 6:**
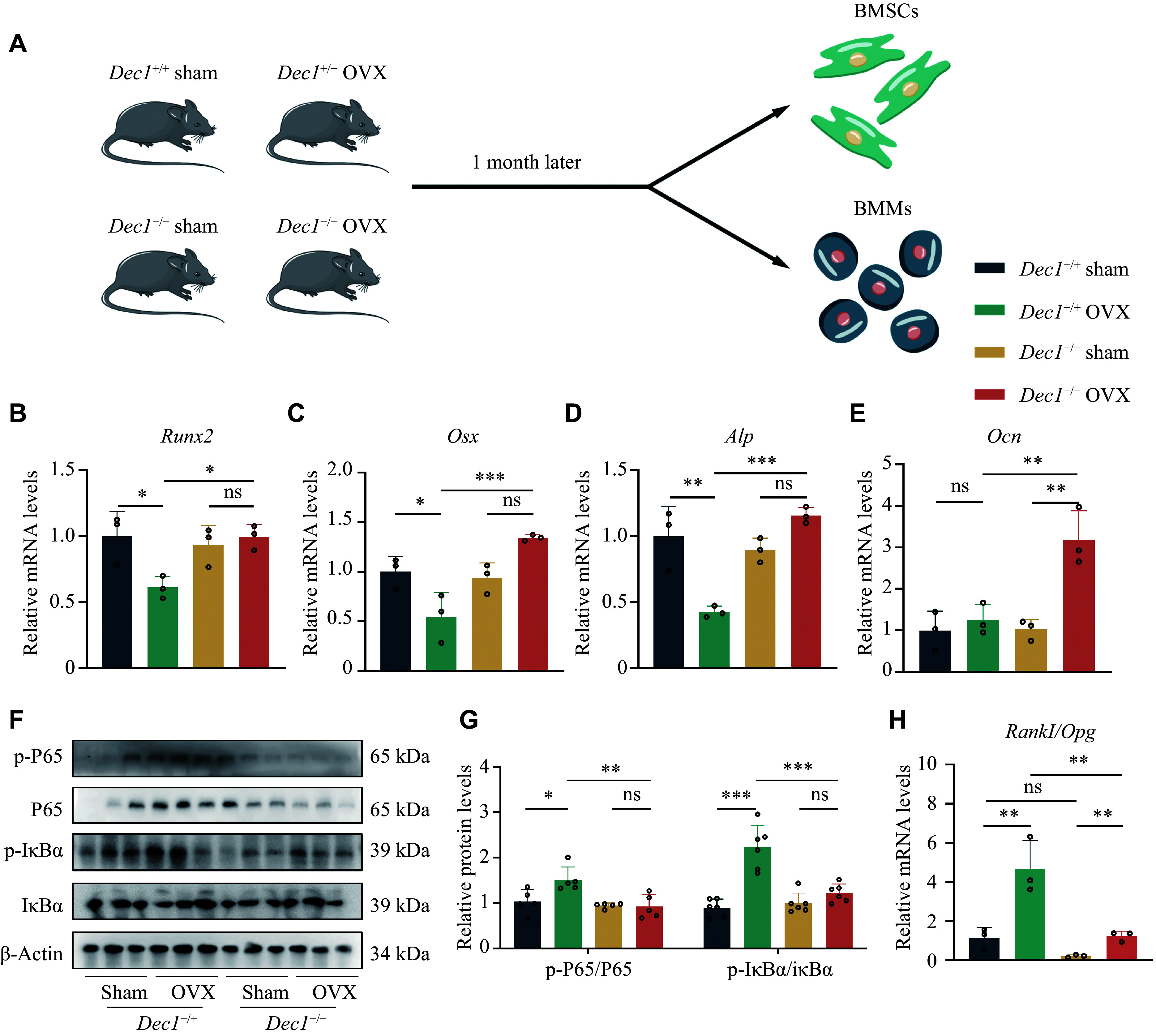
DEC1 deficiency attenuated the NF-κB activation but increased osteoblast activity and the *Rankl/Opg* ratio in BMSCs from the two genotypes of the OVX mice. A: The flow chart of isolating BMSCs and BMMs from the two genotypes of the OVX mice ( *n* = 5 in each group). B–E: The mRNA levels of *Runx2*, *Osx*, *Alp*, and *Ocn* in BMSCs from the two genotypes of the mice ( *n* = 3 in each group). F and G: The protein levels of p-P65, P65, p-IκBα, and p-IκBα in BMSCs from the two genotypes of the mice by Western blotting ( *n* = 5 in each group). H: The mRNA levels of *Rankl*/ *Opg* in BMSCs from the two genotypes of the mice ( *n* = 3 in each group). Data are presented as mean ± standard deviation and analyzed using two-way ANOVA followed by Tukey's honestly significant difference tests. ^*^ *P* < 0.05, ^**^ *P* < 0.01, ^***^ *P* < 0.001, and ^ns^ *P* > 0.05. Abbreviations: DEC1, differentiated embryo-chondrocyte expressed gene 1; RANKL, receptor activator of nuclear factor kappa-B ligand; OPG, osteoprotegerin; OVX, ovariectomy.

To determine the effect of DEC1 in BMSCs and BMMs on bone loss induced by OVX, we isolated and cultured BMSCs and BMMs from both *Dec1* ^+/+^ and *Dec1* ^−/−^ OVX or sham mice and analyzed their osteogenic differentiation capabilities ( ***Fig. 6A***). The results showed that the mRNA levels of genes *Runx2* and *Osx*, which contributed to bone differentiation, as well as the gene alkaline phosphatase ( *Alp*) that promoted bone activity, were significantly decreased in *Dec1* ^+/+^ OVX BMSCs, compared with those in *Dec1* ^+/+^ sham BMSCs, whereas they did not decrease in *Dec1* ^−/−^ OVX BMSCs, compared with those in *Dec1* ^−/−^ sham BMSCs ( ***Fig. 6B***– ***6E***). Moreover, the phosphorylation levels of IκBα and P65 were significantly increased in *Dec1* ^+/+^ OVX BMSCs, compared with those in *Dec1* ^+/+^ sham BMSCs, but did not increase in *Dec1* ^−/−^ OVX BMSCs, compared with those in *Dec1* ^−/−^ sham BMSCs ( ***Fig. 6F*** and ***6G***). Notably, the phosphorylation levels of IκBα and P65 were significantly decreased in *Dec1* ^−/−^ OVX BMSCs, compared with those in *Dec1* ^+/+^ OVX BMSCs, which was consistent with the result of RANKL/OPG *in vivo* ( ***Fig. 3B***). Furthermore, the *Rankl/Opg* ratio was significantly increased in both *Dec1* ^+/+^ and *Dec1* ^−/−^ OVX BMSCs, compared with those in corresponding sham BMSCs, and the increased *Rankl/Opg* ratio was significantly lower in *Dec1* ^−/−^ OVX BMSCs than in *Dec1* ^+/+^ OVX BMSCs ( ***Fig. 6H***). These results indicated that DEC1 deficiency enhanced osteoblast activity while attenuating the NF-κB pathway and increasing the RANKL/OPG ratio in BMSCs from the OVX mice.

### DEC1 deficiency inhibited osteoclast function along with repressing the NF-κB signaling and reducing pro-inflammatory cytokine production in BMMs from the OVX mice

**Figure 7:**
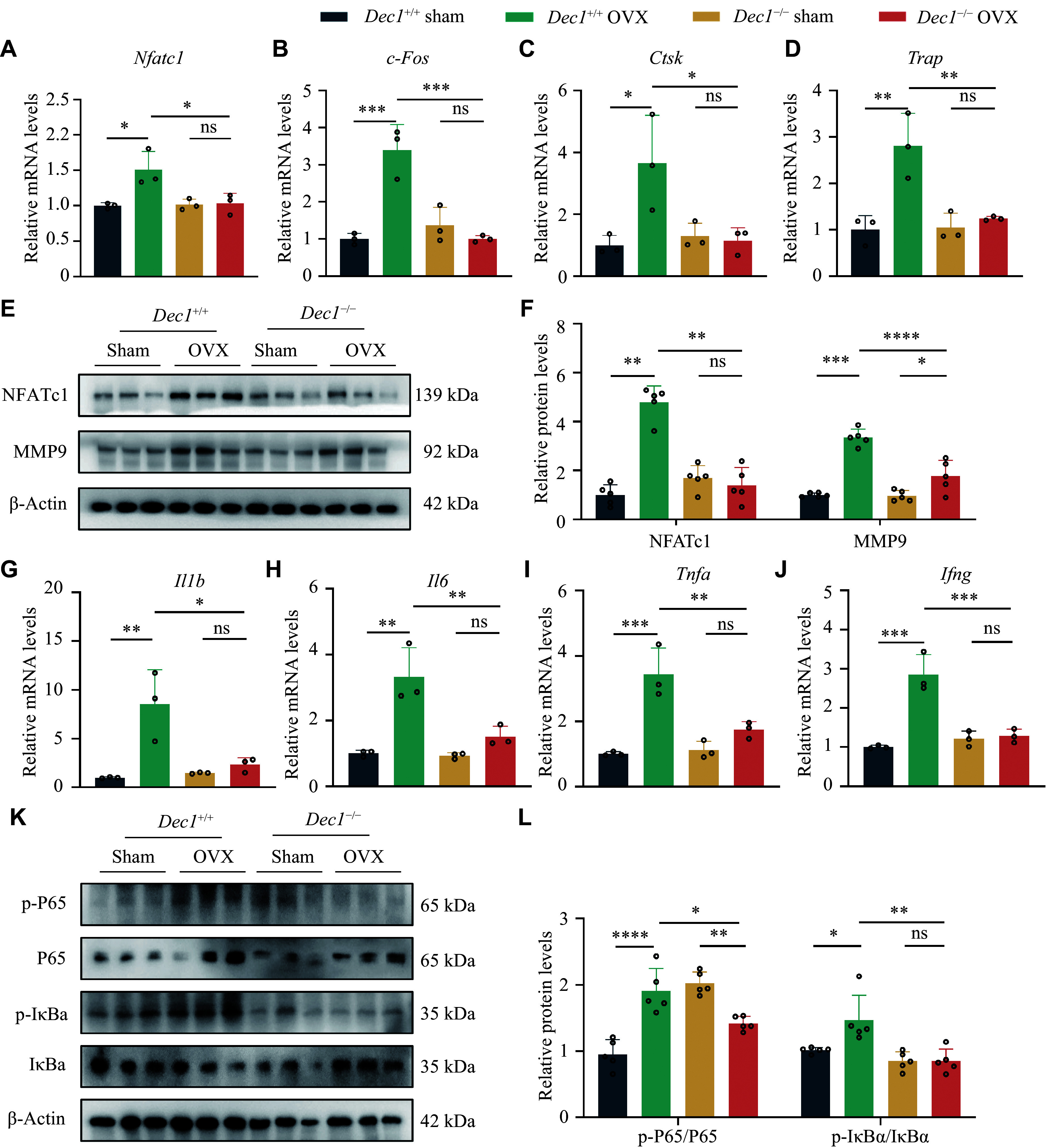
DEC1 deficiency attenuated the osteoclast activation along with decreasing the NF-κB activation and reducing the release of pro-inflammatory cytokines in BMMs from the two genotypes of the OVX mice. A–D: The mRNA levels of *Nfatc1*, *c-Fos*, *Ctsk*, and *Trap* in BMMs from the four groups of mice ( *n* = 3 in each group). E and F: The protein levels of NFATc1, MMP9, p-P65/P65, and p-IκBα/IκBα in BMMs from the four groups of mice ( *n* = 5 in each group). G–J: The mRNA levels of *Il1b*, *Il6*, *Tnfa*, and *Ifng* in BMMs from the four groups of mice ( *n* = 3 in each group). Data are presented as mean ± standard deviation and analyzed using two-way ANOVA followed by Tukey's honestly significant difference tests. ^*^ *P* < 0.05, ^**^ *P* < 0.01, ^***^ *P* < 0.001, ^****^ *P* < 0.0001, and ^ns^ *P* > 0.05. Abbreviations: DEC1, differentiated embryo-chondrocyte expressed gene 1; BMMs, bone marrow-derived macrophages; OVX, ovariectomy; NFATc1, nuclear factor of activated T cells 1; MMP9, matrix metallopeptidase 9.

We further determined the effects of DEC1 in BMMs on the bone loss induced by OVX. We found that the mRNA levels of the genes *Nfatc1* and *c-Fos*, which promoted osteoclast differentiation, *Ctsk* and *Trap*, which were linked to osteoclast activity, were significantly increased in *Dec1* ^+/+^ OVX BMMs, compared with those in *Dec1* ^+/+^ sham BMMs; however, the upregulation of these genes was not observed in *Dec1* ^−/−^ OVX BMMs, compared with those in *Dec1* ^−/−^ sham BMMs ( ***Fig. 7A***– ***7D***). Furthermore, the expression levels of these genes were significantly lower in *Dec1* ^−/−^ OVX BMMs than in *Dec1* ^+/+^ OVX BMMs ( ***Fig. 7A***– ***7D***). Consistent with the mRNA levels, the protein levels of NFATc1 showed a similar trend and pattern ( ***Fig. 7E*** and ***Fig. 7F***). Although the protein levels of MMP9, which was also linked to osteoclast activity, were significantly increased in both *Dec1* ^+/+^ and *Dec1* ^−/−^ OVX BMMs, compared with those in the corresponding sham BMMs, the increased protein levels of MMP9 were significantly lower in *Dec1* ^−/−^ OVX BMMs than in *Dec1* ^+/+^ sham BMMs ( ***Fig. 7E*** and ***7F***).

BMMs are precursor cells to various cell types, and are capable of not only differentiating into osteoclasts but also into macrophages that are crucial components of the immune system and are capable of engulfing pathogens, dead cells, and other debris ^[ 33] ^. During the process of bone remodeling, BMMs influence bone formation and resorption through the release of various cytokines, including growth factors, and pro-inflammatory cytokines ^[ 34– 35] ^. To determine whether the reduced osteoclast function in *Dec1* ^−/−^ OVX BMMs was correlated with inflammation, we measured the mRNA levels of pro-inflammatory cytokines, such as *Il1b*, *Il6*, *Tnfa*, and *Ifng*, in BMMs from the two genotypes of the OVX mice. As a result, the mRNA levels of *Il1b*, *Il6*, *Tnfa*, and *Ifng* were significantly increased in *Dec1* ^+/+^ OVX BMMs, compared with those in *Dec1* ^+/+^ sham BMMs, but not in *Dec1* ^−/−^ OVX BMMs, compared with those in *Dec1* ^−/−^ sham BMMs ( ***Fig. 7G***– ***7J***). Moreover, the mRNA levels of these inflammatory cytokines were significantly lower in *Dec1* ^−/−^ OVX BMMs than in *Dec1* ^+/+^ OVX BMMs. Furthermore, the phosphorylation levels of IκBα and P65 were significantly increased in *Dec1* ^+/+^ OVX BMMs, compared with those in *Dec1* ^+/+^ sham BMMs, but did not increase in *Dec1* ^−/−^ OVX BMMs, compared with those in *Dec1* ^−/−^ sham BMMs ( ***Fig. 7K*** and ***7L***). Notably, the phosphorylation levels of IκBα and P65 were significantly lower in *Dec1* ^−/−^ OVX BMMs than in *Dec1* ^+/+^ OVX BMMs. These results indicated that the DEC1 deficiency might inhibit osteoclast function while repressing the NF-κB signaling and reducing pro-inflammatory cytokine production in BMMs from the OVX mice.

## Discussion

PMOP is a condition affecting postmenopausal women characterized by a decrease in bone mass because of estrogen deficiency. The underlying mechanism involves the direct effects of estrogen on osteoblasts and osteoclasts, which in turn affects bone homeostasis. Furthermore, estrogen deficiency leads to an increase in inflammatory factors in the body, significantly contributing to PMOP ^[ 28] ^. Studies have reported that DEC1 may be associated with immune response ^[ 22] ^. The immune checkpoint therapy upregulated the transcription factor DEC1 in tumor antigen-specific CD8 ^+^ and CD4 ^+^ T cells, while DEC1 deficiency inhibited immune checkpoint therapy-induced macrophage transformation from M2 to M1 subtype ^[ 36] ^. In addition, DEC1 overexpression increased the expression of pro-inflammatory cytokines, such as TNFα, IL-1β, and IL-6 ^[ 37] ^. Therefore, DEC1 may play a pivotal role in intracellular signaling and the regulation of inflammatory responses.

The current study provided some evidence both *in vivo* and *in vitro* to support that DEC1 deficiency protected against bone loss induced by inflammation. Evidence *in vivo*: (1) The phenotypes of bone loss significantly increased in *Dec1* ^+/+^ OVX mice, compared with those in *Dec1* ^+/+^ sham mice, but no difference was observed between *Dec1* ^−/−^ OVX and *Dec1* ^−/−^ sham mice. Likewise, compared with wild-type mice, DEC1 deficiency reduced the phenotypes of bone loss induced by OVX in mice. (2) The osteogenesis-related proteins, such as β-catenin and RUNX2, significantly decreased in *Dec1* ^+/+^ OVX mice, compared with those in *Dec1* ^+/+^ sham mice, but no difference was found between *Dec1* ^−/−^ OVX and *Dec1* ^−/−^ sham mice. Similarly, DEC1 deficiency inhibited the decreased osteogenesis induced by OVX in the mice. (3) The bone destruction-related proteins, such as NFATc1, c-Fos, and CTSK, as well as the RANKL/OPG ratio, were significantly increased in *Dec1* ^+/+^ OVX mice, compared with those in *Dec1* ^+/+^ sham mice, but no difference was observed between *Dec1* ^−/−^ OVX and *Dec1* ^−/−^ sham mice. In the same way, DEC1 deficiency decreased the increased bone destruction induced by OVX in the mice. (4) The serum levels of IL-1β and IL-6 were significantly lower in *Dec1* ^−/−^ OVX mice than those in *Dec1* ^+/+^ OVX mice, implying that DEC1 deficiency inhibited the OVX-induced serum levels of IL-1β and IL-6. In conclusion, osteogenic capacity was protected, whereas osteoclastogenic capacity was inhibited in *Dec1* ^−/−^ OVX mice. Likewise, the release of pro-inflammatory cytokines was significantly inhibited in *Dec1* ^−/−^ OVX mice.

The OVX mice model mimics PMOP through the combined effects of multiple cell types ^[ 5] ^. Evidence *in vitro*: (1) DEC1 expression levels were decreased in BMSCs but increased in BMMs from OVX-induced *Dec1* ^+/+^ mice. (2) Consistent with the *in vivo* results, DEC1 deficiency increased the expression levels of osteogenesis-related genes, such as *Runx2*, *Osx*, *Alp*, and *Ocn*, but decreased the *Rankl*/ *Opg* ratio that was related to osteoclasts in BMSCs induced by OVX. Whereas DEC1 deficiency decreased the osteoclast-related genes, such as *Nfatc1*, *c-Fos*, *Ctsk*, and *Trap*, along with the decreased expression of pro-inflammatory cytokines, such as *Il1b*, *Il6*, *Tnfa*, and *Ifng*, in BMMs induced by OVX, the differential expression of DEC1 in BMSCs and BMMs of *Dec1* ^+/+^ OVX mice prompted us to further consider the following. *In vitro* experiments demonstrated that OVX caused the decreased expression levels of DEC1 in *Dec1* ^+/+^ BMSCs that had the potential for osteogenic differentiation, downregulating the osteogenic capacity. This finding was consistent with our previous study ^[ 25] ^. In contrast, OVX induced the increased expression of DEC1 in *Dec1* ^+/+^ BMMs that possessed potential for osteoclast differentiation, leading to the increased secretion of pro-inflammatory cytokines, such as IL-1β and IL-6. The increased pro-inflammatory cytokines have been found to inhibit osteogenesis on one hand ^[ 38– 39] ^, and promote osteoclastogenesis on the other hand ^[ 39– 40] ^. Conversely, *Dec1* knockout resulted in a significant inhibition of pro-inflammatory cytokine secretion, thereby rescuing the imbalance in bone remodeling in OVX mice. Pro-inflammatory cytokines are released into the bone microenvironment, contributing to the development of osteoblasts and osteoclasts, and in turn affecting bone metabolism and the development of osteoporosis ^[ 41– 42] ^. The NF-κB pathway serves as a critical pathway for pro-inflammatory cytokines (such as IL-1β, IL-6, and TNF-α) ^[ 43] ^. The phosphorylation of IκBα activates NF-κB that mediates cytokine release ^[ 44– 45] ^. To investigate the role of DEC1 in bone loss induced by OVX, we detected the DEC1 expression in both BMSCs and BMMs from *Dec1* ^+/+^ OVX mice and the phosphorylation levels of IκBα and P65 in both BMSCs and BMMs from the two genotypes ( *Dec1* ^+/+^ and *Dec1* ^−/−^) of OVX mice. As a result, we found that the phosphorylation levels of IκBα and P65 were significantly increased in *Dec1* ^+/+^ OVX mice, compared with those in *Dec1* ^+/+^ sham mice, but DEC1 deficiency did not elevate the phosphorylation levels of IκBα and P65 in either BMSCs or BMMs induced by OVX, implying that the upregulation of pro-inflammatory cytokines, such as IL-1β, IL-6, and TNF-α induced by OVX was mediated by the increased activation of the NF-κB pathway. DEC1 deficiency inhibited the NF-κB pathway induced by OVX, thereby reducing cytokine levels. Consequently, the reduced cytokines attenuated the decrease of osteogenesis and the increase of osteoclastogenesis induced by OVX. These results were further supported by the findings that the IKK/NF-κB pathway regulated the osteogenesis and osteoclastogenesis *in vivo* and *in vitro* ^[ 46] ^.

However, there are certain limitations to the current study. First, we used global rather than conditional knockout mice to construct the OVX model. Therefore, we isolated BMSCs and BMMs from *Dec1* ^−/−^ OVX mice and *Dec1* ^+/+^ OVX mice, respectively, to determine the differential role of DEC1 in osteoblasts and osteoclasts. Second, the effects of DEC1 on the NF-κB pathway in BMSCs and BMMs remain to be demonstrated in future studies.

In summary, DEC1 deficiency inhibits the NF-κB pathway induced by OVX, thereby reducing the levels of cytokines, such as IL-1β and IL-6, and subsequently inhibits the decrease of osteogenesis and the increase of osteoclastogenesis caused by OVX. Taken together, DEC1 deficiency protects against bone loss induced by OVX by inhibiting inflammation. These findings provide a novel understanding of postmenopausal osteoporosis development and may offer potential avenues for developing disease intervention strategies.

## SUPPLEMENTARY DATA

Supplementary data to this article can be found online.
