# Supplementary material for: DEC1 deficiency protects against bone loss induced by ovariectomy by inhibiting inflammation
Source: J Biomed Res. 2024 May 29;38(6):613–27. doi: 10.7555/JBR.38.20240069 (PMC11629160; doi:10.7555/JBR.38.20240069)
Supplement: Supplementary file 1 — Supplementary data to this article can be found online. [file jbr-38-6-613-S1.pdf]

Available online at www.jbr-pub.org.cn

Open Access at PubMed Central

Journal of Biomedical Research, 2024 38(6): 1–1

Supplementary Data

JBR

DEC1 deficiency protects against bone loss induced by ovariectomy by inhibiting inflammation

Lan Lin△, Zhiyi Qiang△, Kaiao Chen, Ying Huo, Wei Liu, Jian Yang✉

Department of Pharmacology, Nanjing Medical University, Nanjing, Jiangsu 210066, China.

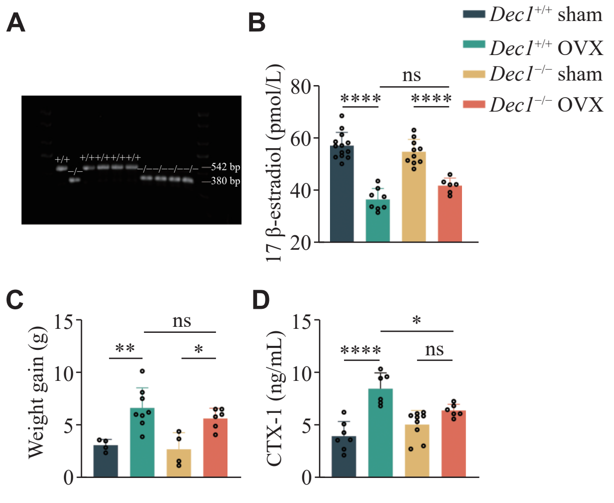

Supplementary Fig. 1 **The phenotypes of the two genotypes (*Dec1*+/+ and *Dec1*−/−) of the OVX mice.** A: Genotype identification of mice. Tail DNA derived from *Dec1*+/+ and *Dec1*−/− mice was extracted and analyzed by using PCR for genotype identification. The protocol, primers, and reagents were provided by RIKEN BioResource Center. The genotype of *Dec1*+/+ and *Dec1*−/− mice is shown in the figure (n = 5 in each group). B: Serum estradiol was analyzed by ELISA in each group of mice (n = 6–13 in each group). C: Body weight in the two genotypes (*Dec1*+/+ and *Dec1*−/−) of the OVX mice (n = 5–8 in each group). D: The serum CTX-1 concentration was analyzed by ELISA in each group of mice (n = 6–9 in each group). Data are presented as mean ± standard deviation and analyzed using two-way ANOVA followed by Tukey's honestly significant difference tests. $^{*}P < 0.05$, $^{**}P < 0.01$, $^{****}P < 0.0001$, and $^{ns}P > 0.05$. Abbreviations: OVX, ovariectomy; CTX-1, carboxy-terminal cross-linked telopeptide of type 1 collagen; ELISA, enzyme-linked immunosorbent assay

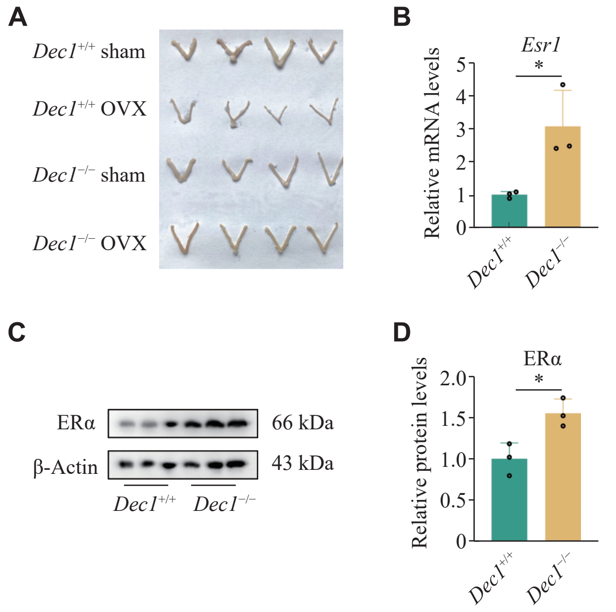

Supplementary Fig. 2 **The size of the uterus and the mRNA levels of *Esr1* and the protein levels of ERα in the uterus of two genotypes of the mice.** A: The size of the uterus in the genotypes (*Dec1*+/+ and *Dec1*−/−) of the OVX mice (n = 4 in each group). B: The mRNA expression levels of *Esr1* in the uterus of the two genotypes (*Dec1*+/+ and *Dec1*−/−) of the mice were detected by real-time PCR (n = 3 in each group). C and D: The protein levels of ERα in the uterus of the two genotypes (*Dec1*+/+ and *Dec1*−/−) of the mice by Western blotting (n = 3 in each group). Data are presented as mean ± standard deviation and analyzed by Student's *t*-test. $^{*}P < 0.05$. Abbreviations: OVX, ovariectomy; Esr1 and ERα, estrogen receptor 1.

△These authors contributed equally to this work.

✉Corresponding author: Jian Yang, Department of Pharmacology, Nanjing Medical University, 101 Longmian Road, Nanjing, Jiangsu 211166, China. E-mail: jianyang@njmu.edu.cn.

Received: 20 March 2024; Revised: 28 April 2024; Accepted: 06 May 2024; Published online: 29 May 2024

CLC number: R58, Document code: A

The authors reported no conflict of interests.

This is an open access article under the Creative Commons Attribution (CC BY 4.0) license, which permits others to distribute, remix, adapt and build upon this work, for commercial use, provided the original work is properly cited.

© 2024 by Journal of Biomedical Research.

https://doi.org/10.7555/JBR.38.20240069
